# 2D-fluoroscopic based navigation for Gamma 3 nail insertion versus conventional procedure- a feasibility study

**DOI:** 10.1186/1471-2474-14-74

**Published:** 2013-02-28

**Authors:** A Wilharm, I Marintschev, G O Hofmann, F Gras

**Affiliations:** 1 Department of Trauma-, Hand- and Reconstructive Surgery, University Hospital Jena, Erlanger Allee 101, Jena 07747, Germany

**Keywords:** Femoral fracture, Navigation, Gamma nail, Geriatric traumatology, Computer assisted surgery, Fluoroscopic navigation, Proximal femoral nailing, Cephalomedullary nail, Trochanteric fracture, Radiation exposure

## Abstract

**Background:**

Intramedullary nailing is a standard surgical procedure for fixation of proximal femoral fractures, but is associated with considerable radiation exposure for controlling the implant placement, due to the percutaneous insertion technique.

The aim of this study was the evaluation of potential benefits of 2D-fluoroscopic based navigation focused on the reduction of radiation exposure, a decrease of procedure time, as well as an increase of accuracy for Gamma3 nail insertions.

**Methods:**

Twenty randomized Gamma3 nail insertions were performed in non-fractured synthetic femora according to the manufactures operation guidelines (group I) or with use of a 2D-fluoroscopic based navigation system (group II). Time of different steps of the procedure and the radiation exposure were measured, as well as the accuracy evaluated in postoperative CT scans.

**Results and discussion:**

All Gamma3 nails were placed without any technical problems. Independent of the used procedure, the overall operating time (group I: 584 ± 99.2 sec; group II: 662 ± 64.9 sec; p=0.06) and accuracy of the final nail-positions were equivalent, but the radiation exposure was significantly reduced (92% reduction in fluoroscopic images and 91% reduction in fluoroscopic time, p< 0.01), using the 2D fluoroscopic based navigation procedure.

**Conclusions:**

2D-fluoroscopic based navigation for Gamma3 nail insertion facilitates a relevant reduction of radiation exposure with equivalent accuracy of the final implant position and no prolonged operating time. This promising procedure modification is independent of different cephalomedullary implant manufacturers and specific implant designs, but needs to be evaluated in further clinical settings.

## Background

Proximal femoral fractures are the second most common fracture in older patients and accounted for 100,927 operative procedures 2009 in Germany. 85% of the patients are over 70 years of age and 72% complain of pre-existing morbidities (ASA≥3) [1], making the operative management more demanding. A short surgery time and use of less invasive approaches are the two key-parameters to reduce surgery related morbidity. Both are addressed by intramedullary nailing of proximal femoral fractures, but are accompanied by increased fluoroscopy time to control the implant placement [2,3]. This is a relevant drawback, especially for the operating team with daily exposure to radiation [4,5].

The aim of this study was the evaluation of potential benefits of 2D-fluoroscopic based navigation for Gamma3 nail insertions focused on the reduction of radiation exposure, a decrease of procedure time, as well as an increase of accuracy defined by an optimal placement of the hip screw in the femoral head, based on the tip apex distance [6].

## Methods

**Figure 1:**
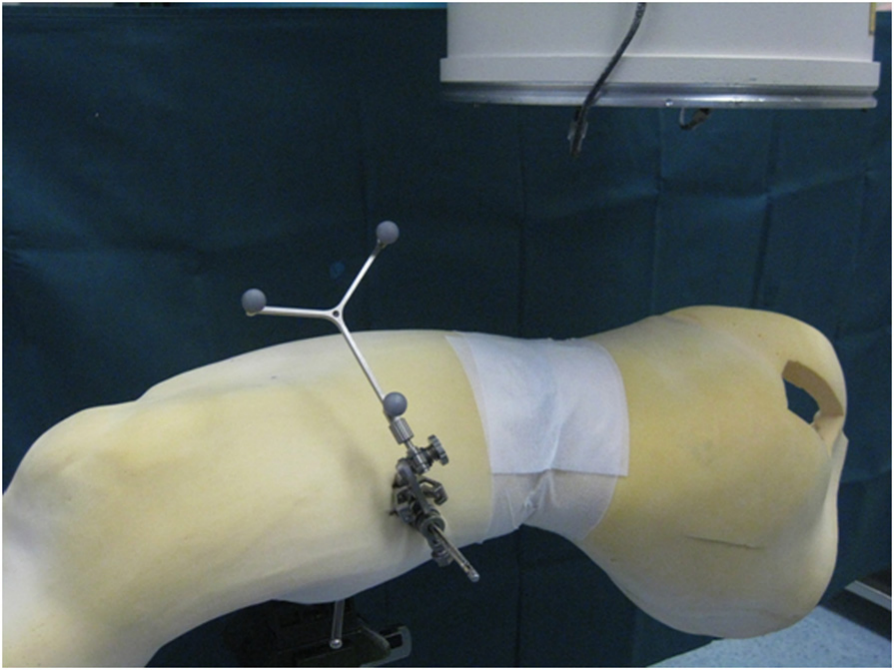
Foam-coated synthetic femur with mounted reference base.

In a standardized experimental setting 20 Gamma3 nails were inserted in non-fractured synthetic femora (Sawbones, Malmö, Sweden) covered in a foam envelope, simulating the soft tissue (Figure 1). In the control group (I) ten procedures were performed in the standard technique according to the manufacturers operation guidelines (Stryker, Duisburg, Germany).

**Figure 2:**
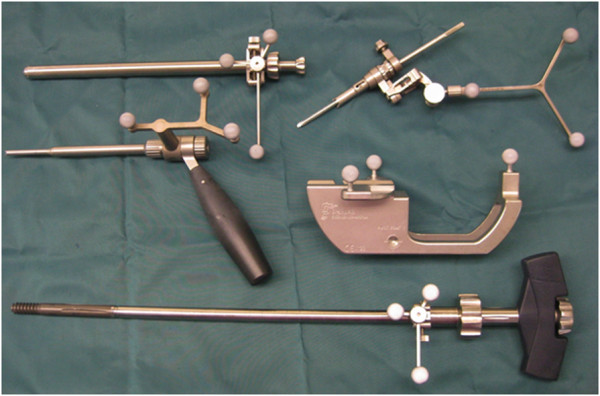
Instruments for the navigation-assisted operation.

**Figure 3:**
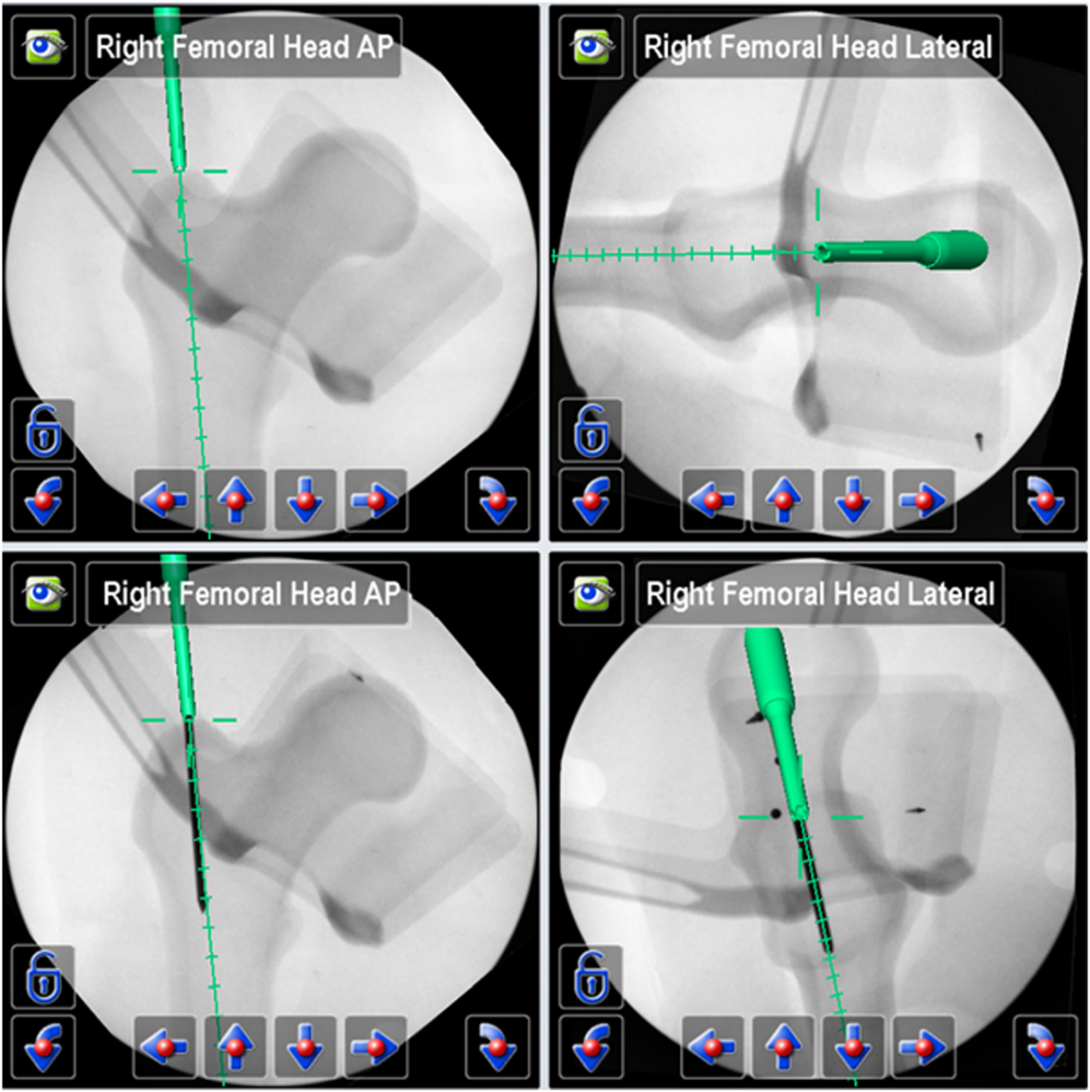
**Navigation step 1** → **Insertion of guide wire (above), virtual projection with guide wire (below).**

**Figure 4:**
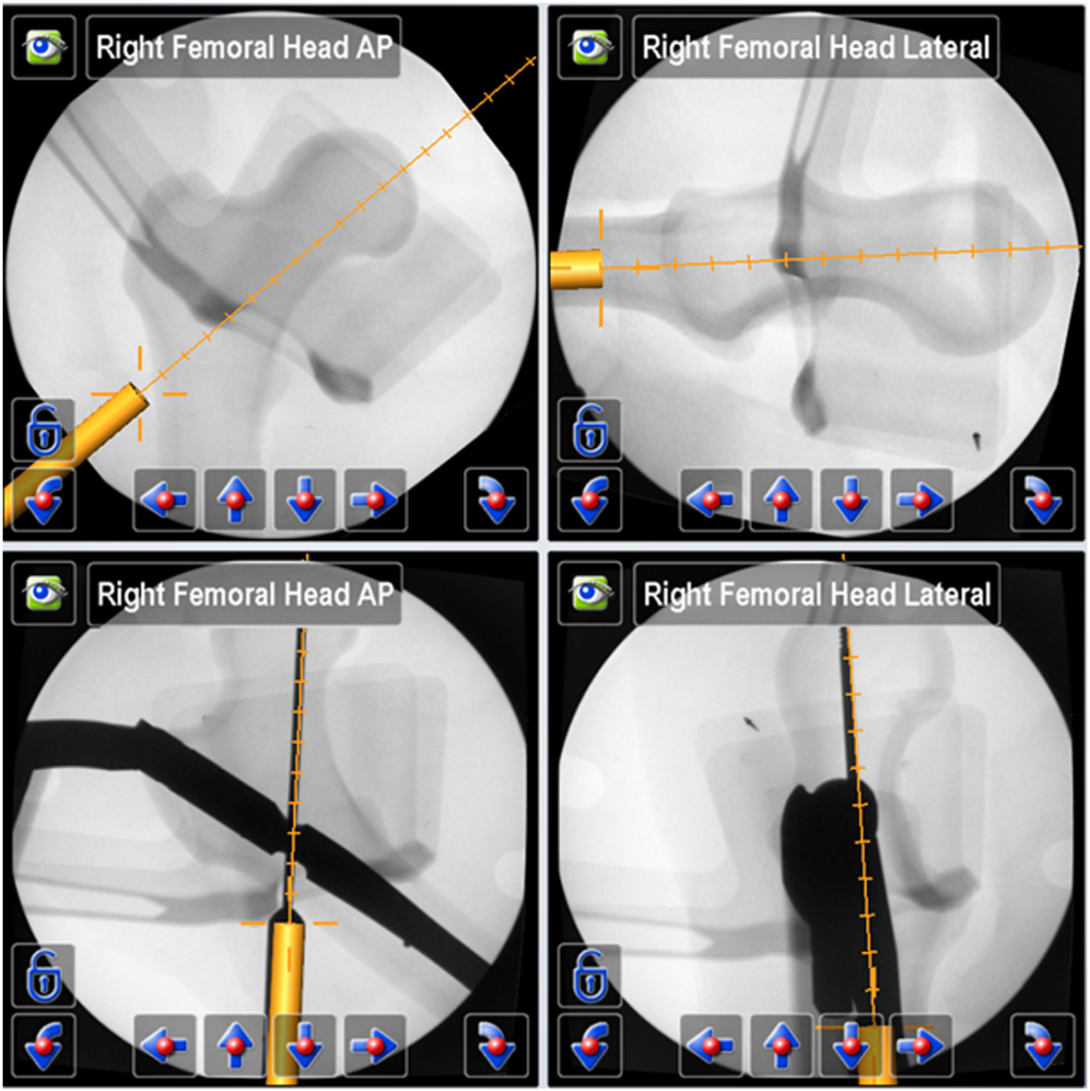
Navigation steps 2 and 3 → Orientation of the intramedullary nail for ideal placement of a guide wire for the femoral neck screw (above), virtual projection with guide wire (below).

**Figure 5:**
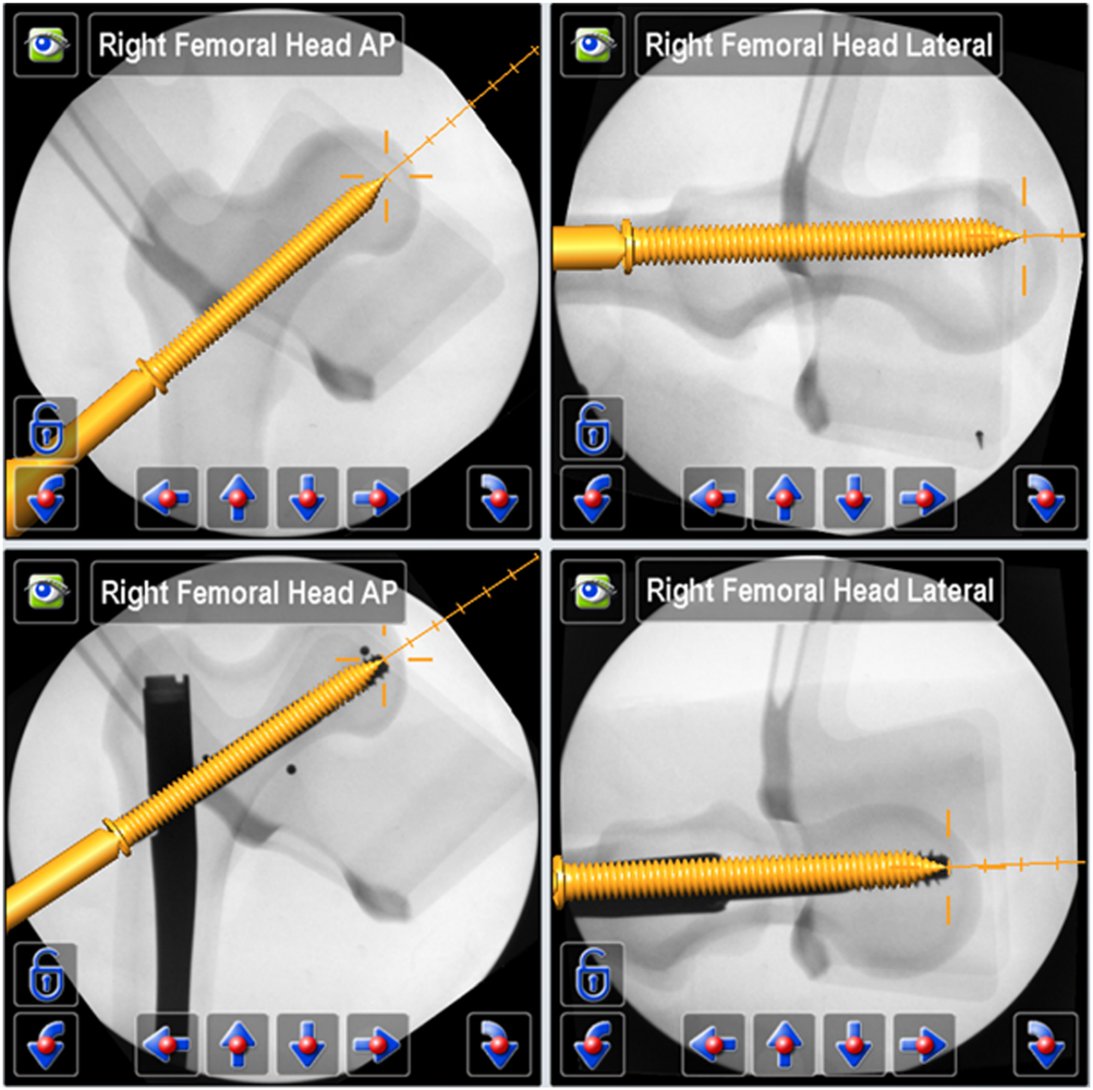
Navigation step 4 → Insertion of a femoral neck screw (above), virtual projection with the femoral neck screw (below).

In the test group (II) ten procedures were performed, using a 2D-fluoroscopic based navigation procedure (VectorVision Trauma 3.0, Brainlab, Feldkirchen, Germany) for the following steps of nail insertion (Figures 2, 3, 4 and 5, Table 1):

1) determination of the right entry point of the nail (at the junction of the anterior third and the posterior two-thirds of the greater trochanter in the axial view and on the tip itself in the ap view)

2) control of the right insertion depth of nail

3) adjustment of an optimized guide wire position for the femoral neck screw insertion

4) control of the insertion depth for femoral neck screw

**Table 1:** Surgical sequence (surgical steps only required only for assisted navigation in italics, navigation-assisted surgical steps underlined, (*) can be done before or parallel to the operation to save time), duration of operation, number of intraoperative X-ray images, necessary fluoroscopy time, significance level p<0.01

**Surgical step**	**Op. time conventional [sec]**	**Op. time navigation-assisted [sec]**	**Significance**	**X-ray images conventional [n]**	**X-ray images navigation-assisted [n]**	**Significance**	**X-ray time conventional [sec]**	**X-ray time navigation-assisted [sec]**	**Significance**
*Instrument calibration* (*)		60.2 ± 9.7							
*Fixation of dynamic reference base*		53.9 ± 5.5							
*Image acquisition*		78.4 ± 27.8			2.6 ± 1.1			1.5 ± 0.5	
Guide wire placement for nail insertion	161.8 ± 71.4	79.4 ± 21.6	✓	12.4 ± 5.9		✓	6.6 ± 3.5		✓
Drilling	31.7 ± 5.8	39.7 ± 13.5							
Nail insertion	67.4 ± 18.6	44.0 ± 8.7	✓	5.0 ± 2.5		✓	2.8 ± 1.7		✓
Guide wire placement for the femoral neck screw	122.1 ± 57.4	31.6 ± 7.4	✓	9.7 ± 3.4		✓	4.8 ± 2.0		✓
Drilling for femoral neck screw	22.5 ± 4.8	23.6 ± 5.8							
*Calibration of femoral neck screw* (*)		81.6 ± 32.4							
Femoral neck screw insertion	74.7 ± 8.8	57.5 ± 9.8	✓	5.9 ± 1.1		✓	3.2 ± 0.6		✓
Distal locking	77 ± 5.1	77.5 ± 5.4							
Control X-ray	26.3 ± 8.2	34.6 ± 9.5							
Total	583.5 ± 99.2	662 ± 64.9		33 ± 8.8	2.6 ± 1.1	✓	17.4 ± 4.6	1.5 ± 0.5	✓

For the navigation procedure only two fluoroscopic images (antero-posterior and axial view) were acquired using a standard image intensifier (Ziehm Vision, Nuremberg, Germany) and a x-spot® for the navigation specific registration procedure [7]. However prior to that, a dynamic reference base had to be rigidly fixed with a 5mm Schanz screw at the distal femur.

Furthermore it was necessary to calibrate a 3.0 mm drill sleeve for the navigated determination of the nail entry point, the standard femoral neck screw sleeve for the navigated control of the alignment and length of the neck screw, as well as the tip of the neck screw to control their insertion depth.

After randomization by drawing lots all operations were performed by only one surgeon, experienced in both - conventional and navigation-assisted - insertion techniques. To prevent a visual and proprioceptive memory effect [8], the femoral bones were clamped in different axial and rotational positions.

The duration of each surgical step, the fluoroscopy time, as well as the number of fluoroscopy images were recorded (Table 1). The nail positions were evaluated postoperatively by CT scans (4.X, GE Medical Systems, Milwaukee, USA) measuring the tip apex distance (TAD) and the screw position in the femoral neck [6]. The aim was a TAD less than 25 mm and a centrocaudal position of the screw in the femoral neck.

For statistical analysis an excel data sheet and SPSS Version 17.0 (SPSS Inc., Chicago, IL, USA) were used. The significance level of the used t-test was p < 0.01 (Table 1).

## Results and discussion

All procedures in both groups were performed without any technical problems. Statistically significant differences were measured for the following two parameters:

1. First pass accuracy of guide wire insertion. The determination of the optimized nail insertion point and guide wire placement for the femoral neck screw required respectively 4.0 and 4.1 attempts in the conventional group, whereas only 1.0 attempt was performed under navigated guidance for both.

2. Radiation exposure. Using the standard operating procedure, on average of 33 ± 8.8 fluoroscopic images with a fluoroscopy exposure time of 17.4 ± 4.6 seconds compared to only 2.6 ± 1.1 images with a fluoroscopy exposure time of 1.5 ± 0.5 seconds were necessary for the navigated Gamma3 nail insertion.

No significant difference was observed for the overall procedure time in our experimental set up (group I: 584 ± 99.2 seconds vs. group II 662 ± 64.9 seconds). However evaluating the different steps of the procedure, a reduction for all navigated guide wire placements (time saving: 51% to determine the insertion point of nail, 74% to align the femoral neck screw position) and the controls of implant insertion (time saving: 35% for the nail; 23% for the femoral neck screw) were evident.

This is mainly caused by the virtual navigated control in two acquired images only, whereas in the conventional technique several images in two orthogonal projections are mandatory for each step (both guide wires, as well as the nail and femoral neck screw insertion).

However this reduction of operative time was neutralized by additional navigation specific steps, like the calibration of instruments, the placement of a dynamic reference base and the acquisition of the two fluoroscopic images for the navigated procedure.

Using an optimized navigation workflow with calibration of instruments before the operating procedure will be started or parallel to the operation by the nurse, a further reduction of operating time by 10,8% can be expected (group I: 584 ± 99.2 seconds vs. group II 520 ± 48,8 seconds).

In all steps necessary for both operating procedures, that are independent of fluoroscopic or navigated control (like cannulated drillings to open the nail insertion point (p= 0,15) and to prepare the femoral neck screw canal (p= 0,48), as well as placement of the distal locking screw (p= 0,78)), no significant differences were observed.

All Gamma3 nails in both groups were accurately placed without any misplacement. No differences were observed for the mean tip - apex - distance and the position of the femoral neck screw in the postoperative CT scans.

The additional fluoroscopic images - only acquired after each guide wire placement to evaluate the navigation accuracy - showed an exact congruity of the plan on the navigation system display and the final guide wire position (Figure 3, Figure 4).

The use of 2D-fluoroscopic based navigation decreases the radiation exposure for the patient and the operating team substantially during Gamma3 nail insertion. For all operating steps, that need fluoroscopic control of guide wire or implant placement, a reduction in procedure time was observed, but was equalized by additional navigation specific steps, like calibration of instruments, fixation of the dynamic reference base and acquisition of the two fluoroscopic images. The final accuracy for the 20 nail insertions was similar and independent of the conventional or navigated technique used.

Nevertheless, surgical navigation seems to be a promising procedure for the insertion of Gamma3 nails, as reported in this study for the first time.

Especially, the 93% reduction of fluoroscopic images during the nail insertion is one of the most important benefits, as reported for navigated screw fixations in most experimental and clinical studies (87%), as well as the navigated insertion of dynamic hip screws (75%) [9,10].

Whereas the patient is only once exposed to radiation only once, the operating team and predominantly the hands of the surgeon are in close proximity to the radiation beam every day [4]. Furthermore, the frequent movements of the c-arm in the antero-posterior and axial projections during the standard procedure may jeopardize the sterility of the operating field and is associated with increased operating time.

One drawback of most navigation procedures is the prolonged operating time, leading to minor acceptance of most surgeons. Especially for surgical procedures in older patients with several comorbidities this is of relevant concern.

In contrast to the recently published studies (Table 2), no increase of procedure time was observed by use of a navigation system compared to the standard technique. A potential source for further time savings is the calibration of instruments before starting the procedure, as well as faster surface referencing with reference base fixation by velcro-tapes and stretch films, which is under evaluation for accuracy at the moment.

**Table 2:** Studies of navigation-assisted surgery of the proximal femur

	**Type of Study**	**Application**	**navigation-assisted *vs. *conventional**		
			**Op. time**	**Fluor. Time**	**Precision**
Kendoff et al.	exp.	FN screws	**>**	**<**	**=**
2006, Unfallchirurg [14]					
Kendoff et al.	exp.	FN screws	**>**	**<**	**=**
2006, Comp aided surg					
Hamelinck et al.	exp.	FN screws	**=**	**>**	**=**
2006, CORR [15]					
Liebergall et al.	clin.	FN screws	**=**	**=**	**>**
2006, JBJS Br. [16]					
Chong et al.	clin.	DHS	**>**	**<**	**<**
2006, Injury [3]					
Müller et al.	exp.	FN screws	**>**	**<**	**=**
2011, Unfallchirurg [9]					
Beckmann et al.	exp.	FN drilling	**=**	**<**	**>**
2007, Orthopädie [17]	clin.		**>**	**<**	**=**
Müller et al.	exp.	DHS	**>**	**<**	**=**
2011, Rofo [10]					
**Own data /** **Wilharm et al.**	**exp.**	**Gamma3**	**≤**	**<**	**=**

Surprisingly the final precision for the Gamma3 nail insertion did not differ in both groups, but using the navigated approach a first pass accuracy was observed for the placement of guide wires (compared to 4 attempts for determination of the nail insertion point and 4.1 attempts for the cannulated femoral neck screw insertion). Various studies have identified the “first pass accuracy” of guide wire placement as a relevant advantage of navigation procedures [3,11]. Beside a reduction of procedure time, a reduced incidence of incorrect drillings prevents weakening of the lateral cortex and of the cancellous bone in the femoral neck and thereby may reduce the risk for displacement of the femoral neck screw [5,12]. In addition, after multiple unsuccessful attempts of guide wire placement the risk of guide wire slipping into the old drill canals increases with each correction manoeuvre.

Some limitations of the study have to be considered before the navigated procedure can be used in clinical settings:

Firstly, an alternative non-invasive fixation of the dynamic reference base should be preferred to prevent associated morbidities, such as iatrogenic fractures, heterotopic ossifications and soft tissue damage, as reported for different applications of navigation in orthopaedic surgery [13].

Secondly, unintended movements of the femoral head fragment in relation to the femoral shaft after image acquisition will not be detected in the navigated technique. Therefore we recommend additional fluoroscopic images, first to control the final guide wire placement for the femoral neck screw and second during the femoral neck screw insertion to visualize a potential fracture displacement by femoral head rotation.

Thirdly, the procedures were performed by a surgeon experienced in navigation surgery and therefore the measured parameters do not represent the well-known learning curve of inexperienced surgeons, as reported in other studies [9].

## Conclusions

The reported 2D-fluoroscopic based navigation procedure is a promising universal approach for several proximal femur nails due to the independence of different manufacturers and specific implant designs.

Beside an important reduction of radiation exposure, operating-time savings may be achieved with further modification of the navigation workflow and therefore could receive further evaluation in clinical studies.

## Competing interests

No funding was received to support this study and the authors declare that they have no conflict of interest.

## Authors’ contributions

The idea goes back to AW. He also wrote the first conception, carried out the operations and wrote the article. IM made substantial contributions to the conception of the study, helped to perform the analysis of the data and was involved in drafting and revising the manuscript. GH is the head of the department and checked the conception of the study, was involved in the interpretation of the data and was involved in revising the manuscript. FG made substantial contributions to the conception of the study, assisted during the operations, performed the analysis of the data and helped to write the article. All authors read and approved the final manuscript.

## Pre-publication history

The pre-publication history for this paper can be accessed here:

http://www.biomedcentral.com/1471-2474/14/74/prepub
